# Aloe Vera Gel and Cesarean Wound Healing; A Randomized Controlled Clinical Trial

**DOI:** 10.5539/gjhs.v7n1p203

**Published:** 2014-08-31

**Authors:** Zahra Molazem, Fatemeh Mohseni, Masoumeh Younesi, Sareh Keshavarzi

**Affiliations:** 1 Community Based Nursing & Midwifery Research Center, Faculty of Nursing and Midwifery, Shiraz University of Medical Sciences, Shiraz, Iran; 2 Shiraz University of Medical Sciences, Shiraz, Iran; 3 Fellowship of Infertility and Laparoscopic Surgery; 4 Department of Epidemiology Faculty of Health & Nutrition Shiraz University of Medical Sciences, Shiraz, Iran

**Keywords:** aloe vera, caesarean, healing, wound

## Abstract

**Background:**

Failure in complete healing of the wound is one of the probable complications of cesarean. The present study aimed to determine the effectiveness of dressing with aloe vera gel in healing of cesarean wound.

**Methods:**

This prospective randomized double-blind clinical trial was conducted on 90 women who had undergone cesarean operation in Amir-al-Momenin hospital (Gerash, Iran). The participants were randomly divided into two groups each containing 45 patients. In one group, the wound was dressed with aloe vera gel, while simple dressing was used in the control group. Wound healing was assessed 24 hours and 8 days after the cesarean operation using REEDA scale. The data were analyzed through Chi-square and t-test.

**Results:**

The participants’ mean age was 27.56±4.20 in the aloe vera group and 26.62±4.88 in the control group, but the difference was not statistically significant. However, a significant difference was found between the two groups concerning body mass index, heart rate, and systolic blood pressure (P<0.05). Also, a significant difference was observed between the two groups with respect to the wound healing score 24 hours after the operation (P=0.003). After 8 days, however, the difference in the wound healing score was not significant (P=0.283). Overall, 45 participants in the aloe vera group and 35 ones in the control group had obtained a zero score 24 hours after the operation. These measures were respectively obtained as 42 and 41eight days after the operation.

**Conclusion:**

According to the findings of this study, the women are recommended to be informed regarding the positive effects of dressing with aloe vera gel.

## 1. Introduction

The rate of cesarean section has increased in Iran and many other countries in the recent years (Ghaffari et al., 2011). This, in turn, has resulted in a rise in the complications and difficulties caused by the operation (Pallasmaa et al., 2010). Infection of the wound, hematoma, serosa, and wound opening are among the most common complications of cesarean section, resulting in hospitalization of the mother, her repeated referral to doctor for wound drainage and debridement, treatment of the wound for the second time, and a great economic burden imposed on the family (Pallasmaa et al., 2010; Samadi et al., 2010). Hence, wound healing is the main objective after surgical operations or traumatic injuries. Healing of the wound consists of coordinated mending responses that begin after the surgical operations or traumas and leads to reconstruction of the tissue. These responses are closely related to treatments before and after the operations and traumas. Healing process takes place in all wounds gradually. This process includes three phases, namely inflammatory phase, proliferative phase, and maturation phase (Campos, Groth, & Branco, 2008). However, several factors postpone the healing of the wound, including twin delivery, fever, prolonged rupture of the fetal membrane, history of chronic systemic diseases, preeclampsia, obesity, consumption of immunosuppressive drugs, failure in observing sterile precautions before and during the operation, not using prophylactic antibiotics, surgical operation beyond 90 minutes, operation technique, poor homeostasis, type of thread used in subcutaneous repair, and malnutrition (De Vivo et al., 2010; Schneid-Kofman, Sheiner, Levy, & Holcberg, 2005). Sufficient treatment after the operation is effective in healing of the wound. Due to the high costs of chemical treatments and insufficient effects of medications, a tendency to use traditional medicine in healing and prevention of infections has been on the rise. One of the methods used for treatment of wounds is using aloe vera gel.

Aloe vera (Note 1) is a medical plant from the lily family which has attracted the attention of medical scholars in the recent years. This plant is known as a therapeutic plant and has been used for medical purposes for a long time (Sahu et al., 2013). Different parts of this plant have different applications. The thick and transparent gel of the plant, which is stored inside the leaves, is used for treatment of different kinds of wounds. In addition, the yellowish bitter sap on the surface of the leaves has laxative properties and contains antrakoin compounds.

Up to now, several studies have been conducted on the healing effects of aloe vera plant (Sahu et al., 2013). In one study which was conducted on the wound and inflammation of the mucous membrane of the mouth, aloe vera mouthwash was effective in healing of the wound and reducing the inflammation compared to the control group (Mansour, Ouda, Shaker, & Abdallah, 2013). Moreover, this plant’s gel has been reported to be effective in healing of the first and second degree burning wounds without any side effects (Maenthaisong, Chaiyakunapruk, Niruntraporn, & Kongkaew, 2007). Besides, its topical application has been recommended for accelerating the healing process of dermic injuries, such as burns, wounds, frostbites, inflammations, and cutaneous infections (Joseph & Raj, 2010). Overall, it can be concluded that aloe vera gel is an appropriate and economical dressing for a variety of wounds. Nonetheless, contradictory results have been obtained regardingthe effectiveness of this plant. For instance, one study reported delay in healing of the wounds after application of this plant (Schmidt & Greenspoon, 1991).

Up to now, no clinical trials have been performed on the effects of aloe vera gel on cesarean wound healing in Iran. Moreover, considering the high rate of cesarean section in Iran and its resultant complications, there is an evident need to study the effects of aloe vera gel on healing of cesarean wound.

## 2. Materials and Methods

### 2.1 Study Design and Population

This prospective randomized double-blind clinical trial aimed to assess the effect of aloe vera gel on cesarean wound healing between 23 July and 22 November 2013.

The study mothers were18-36 years old with term pregnancy duration of 37–42 weeks. Considering α=0.05 and power=80%, a 90-subject sample size was determined for the study (45 subjects in each group). The participants were divided into an aloe vera and a control group through block randomization. They underwent cesarean section by the same gynecology surgeon in Amir-al-Momenin Hospital in Gerash, Fars province, Iran.

The inclusion criteria of the study were term pregnancy (37–42 weeks), Body Mass Index (BMI)>29, not having the history of more than two cesarean sections, and being willing to participate in the study. On the other hand, the exclusion criteria of the study were suffering from placenta previa, placental abruption, chorioamnit, meconium discharge, and polyhydramnios, having a history of any disease that could affect wound healing, having abnormal fetus, hospitalization of the fetus in Neonatal Intensive Care Unit (NICU), severe bleeding and need for transfusion, having undergone hysterectomy or myomectomy during the operation, having the history of smoking cigarettes or drug abuse, having membranes rupture before the operation, prolongation of the operation for more than 90 minutes, and not referring to the hospital 8 days after the operation.

### 2.2 Ethics

At first, the participants were provided with enough information about the study procedure and objectives, benefits, nature, and duration of the study. They also signed written informed consents after being ascertained about the confidentiality of their information. They were also ensured that they could withdraw from the study whenever they desired.

A demographic information form was used to acquire the mothers’ information, including their age, occupation, gestational age, number of pregnancies, neonate’s sex, duration of operation, duration of Nil Per Os (NPO) before the operation, height, weight, BMI, blood pressure, heart rate, temperature, and the method of anesthesia. Additionally, the wound healing condition was assessed by the REEDA scale whose reliability and validity have been confirmed previously (Hill, 1990).

REEDA scale is an instrument containing five factors, namely redness, edema, ecchymosis, discharge, and approximation of the two edges of the wound. Each of these factors is given a score between 0 and 3 representing no sign and presence of the sign to the highest degree, respectively. Thus, the total score of the scale ranges from 0 to 15, with higher scores showing weaker wound healing. In order to assess the reliability of the scale, wound healing was checked two times, first by the researcher and then by agynecologist, using the REEDA scale. Afterwards, the findings were compared and their consistency was verified (r=0.79).

The participants were visited by the researcher in the hospital before the operation. After obtaining the written informed consents and collecting the demographic data, the participating patients were introduced to the gynecologist. Then, the gynecologist was provided with the table of random numbers (obtained through block randomization) to divide the patients in an aloe vera gel and a control group. It should be noted that the researcher and the patients were unaware of the type of intervention. Cesarean section was performed successfully on all the participants, with a pfannenstiel skin incision and low transverse cesarean incision in the uterus.

In all the cases, a plastic suture with nylon thread No. 3.0 was used for skin repair.

### 2.3 Intervention

Aloe vera gel was used for the intervention group. In doing so, after washing the aloe vera leaves with antiseptic solution, the leaves were cut and their epidermis was removed using gloves and a sterile knife. Then, the mucilage gel inside the leaves was taken out by the surgeon using sterile gloves, was directly applied to the sutured cesarean wound, and was then dressed with dry gauze. In the control group, on the other hand, dressing of the sutured wounds was done using dry gauze alone. Twenty four hours after the operation, the dressing was removed by the nurses and wound healing was assessed by the researcher using the REEDA scale. The participants were asked to refer to the hospital 8 days later for another examination. On the 8^th^ day, wound healing was assessed and recorded once again using the REEDA scale. Then, the stiches were removed.

A diclofenac suppository was prescribed for all the mothers after their transfer from the operation room. They received 1 gr intravenous cefazolin every 6 hours on the first day after the operation and they were discharged from the hospital 24 hours after the operation. In addition, 500 mg cephalexin capsules were prescribed for every 6 hours in the first 7 days, and 250 mg mefenamic acid capsules were prescribed for every 8 hours in the first 3 days. All the mothers breastfed their neonates immediately after birth and transference to the surgery ward. Bleeding from the surgical site was not detected in any of the participants. Also, none of the subjects had abnormal vaginal bleeding.

All the participants were instructed how to take care of themselves after the operation, including protection of the abdominal wall and the stitches during coughing, taking a bath, prevention of stretching of the stiches during movement, avoiding flatulent food, consumption of liquids and laxative food to prevent constipation, daily consumption of dairy products, fruits, vegetables, and meat, and timely intake of the prescribed medicine.

### 2.4 Statistical Analysis

All the study data were analyzed using the SPSS statistical software (version 19). Chi-square test and independent T-test were used in order to compare the distribution of the qualitative and quantitative demographic variables, respectively. Independent T-test was also used to compare the REEDA scores.

## 3. Results

After the screening phase, 146 women were assessed for eligibility. Out of these women, 97 met the inclusion criteria and were randomly divided into an aloe vera and a control group. However, 7 women were lost to follow up. After all, 90 participants were enrolled into the study (45 in the aloe vera group and 45 in the control group).

The two groups’ demographic characteristics have been presented in Tables 1 and 2. Accordingly, the two groups were homogeneous with respect to age, education level, gestational age, parity, history of abortion, neonate’s sex, method of anesthesia, duration of NPO, operation time, height, weight, diastolic blood pressure, and temperature before and after the operation (P>0.05). However, a significant difference was observed between the two groups regarding BMI, heart rate, and systolic blood pressure (P<0.05).

**Table 1:** Comparison of the means of demographic variables in aloe vera and control groups

Characteristics	Aloe vera mean (SD)	Control mean (SD)	P-value
Age	27.56±4.20	26.62±4.88	**0.333**
Gestational age	38.69±1.10	38.73±1.12	**0.850**
NPO time	8.04±1.36	7.96±1.92	**0.801**
Operation time	39.16±4.36	39.44±4.47	**0.757**
BMI	30.24±1.25	30.80±1.35	0.043*
Systolic blood pressure immediately after the operation (mmhg)	112±10.71	117±11.45	0.025*
Diastolic blood pressure immediately after the operation (mmhg)	75.57±6.65	77.69±6.22	**0.123**
HR	81.91±11.44	76.78±11.51	0.037*
Temperature before the operation (Celsius)	37.06±0.28	37.05±0.25	**0.905**
Temperature immediately after the operation (Celsius)	37.08±0.26	37.08±0.38	**0.961**

Note: Significant at α=0.05.

**Table 2:** Comparison of distribution of qualitative demographic variables in aloe vera and control groups

	Aloe vera N (%)	Control N (%)	P-value
Education (Diploma and higher degrees)	31(68.9)	29(64.4)	**0.404**
Primary gravid	19(42.2)	24(55.8)	**0.112**
Abortion history	4(7.9)	6(13.3)	**0.506**
General anesthesia	39(86.7)	35(77.8)	**0.270**
Neonate’s sex (male)	22(48.9)	27(60)	**0.290**

Note: Significant at α=0.05.

The score of cesarean wound healing based on the REEDA scale was 0.00±0.00 in the aloe vera group and 0.6±1.3 in the control group 24 hours after the operation, and the difference was statistically significant (P=0.003). After 8 days, the REEDA score was 0.11±0.49 in the aloe vera group and 0.29±0.99 in the control group, but the difference was not statistically significant (P=0.283). Twenty four hours after the operation, 45 participants in the aloe vera group and 35 ones in the control group had a zero REEDA score (P=0.001). These measures were respectively obtained as 42 and 41 eight days after the operation (P=0.694) (Tables 3 and 4). It should be noted that none of the intervention group participants presented any specific complications.

**Table 3:** Comparison of the mean scores of cesarean wound healing in aloe vera and control groups

	Aloe vera Mean (SD)	Control Mean (SD)	P-value
REEDA scale 24h post caesarean	0.00±0.00	0.60±1.30	0.003*
REEDA scale 8 days post caesarean	0.11±0.49	0.29±0.99	**0.283**

Note: Significant at α=0.05.

**Table 4:** Comparison of the number (%) of participants with zero cesarean wound healing scores in aloe vera and control groups

	Aloe vera N (%)	Control N (%)	P-value
0 REEDA score 24h post caesarean	45(100)	35(77.80)	0.001*
0 REEDA score 8 days post caesarean	42(93.33)	41(91.11)	**0.694**

Note: Significant at α=0.05.

## 4. Discussion

The findings of this study indicated that aloe vera gel was effective in cesarean wound healing 24 hours post cesarean. The results revealed a significant difference between aloe vera and control groups regarding REEDA score after 24 hours; however, no such difference was observed after 8 days. Of course, the intervention group’s REEDA score was lower compared to the control group on the 8^th^ day. Since the intervention was only performed for 24 hours, longer application of the gel might lead to more desirable results.

Up to now, a large number of studies have been conducted on aloe vera gel as an ameliorative and wound healer, confirming the results of this study. However, none of these studies has investigated the effect of this gel on abdominal surgery wound healing using the REEDA scale (Maenthaisong et al., 2007; Mansour et al., 2013). Aloe vera gel is a mucilage in the inner part of the leaves and has attracted the attention of many researchers because of its different properties, such as anti-inflammatory, antioxidant, antiseptic, anti-microbial, anti-fungal, and anti-bacterial features, as well as its effect on improvement of the immune system (Habeeb et al., 2007; Khan, Tania, Zhang, & Chen, 2010; Sahu et al., 2013).

In most of the previous studies, aloe vera gel was used in form of a processed product, such as cream or gel (Dat, Poon, Pham, & Doust, 2012; Eshghi et al., 2010). In this study, however, the fresh natural gel was used for the first time in order to preserve its effective substances. Some researchers have stated that some of the effective substances of aloe vera gel are wasted in the process of preparation and conservation (Sahu et al., 2013; Yeh, Eisenberg, Kaptchuk, & Phillips, 2003).

In a study with a small sample size (19 patients), application of aloe vera gel was recommended due to its low cost and lack of any specific side-effects (Boroujeni et al., 2009). However, in the study Schmidt and Greenspoon (1991) conducted on the infected and unhealed wounds of cesarean section and gynecological abdominal surgery, aloe vera caused a delay in healing of the wounds (Schmidt & Greenspoon, 1991). This can be due to difference in the study design or the patients’ wounds. They also used a different scale for assessment of wound healing. Furthermore, Oliveira, Soares, and Rocha (2010) applied aloe vera on ischemic wounds, and Shahzad and Ahmed (2013) used it on burn wounds (Oliveira, Soares, & Rocha, 2010; Shahzad & Ahmed, 2013). In both cases, the gel was effective in wound healing, which is consistent with the results of the present study.

In this study, the researchers did their best to eliminate the factors that might affect wound healing. They also employed a large sample size in order to come to reliable results. Moreover, wound healing was assessed by a different individual from the interventionist (intervention was done by a gynecologist, while assessment was performed by the researcher), so that the study was blinded. Cleanness of the wounds and quick absorption of the gel via skin made it possible for the aloe vera gel to have the highest efficiency in wound healing. However, it was not possible to take a biopsy of the wound and make a closer examination of the condition of wound healing. Also, it was not possible to fully control the patients’ nutrition at home, their sanitation, and their amount of movement which could affect wound healing. Yet, the researchers made their attempt to compensate for these limitations by random selection of the samples as well as by identically training the patients.

## 5. Conclusion

The present study showed that aloe vera gel was effective in cesarean wound healing. According to the findings of this study, dermal application of aloe vera gel did not have any side effects and it could consequently be used as an adjunctive treatment to the standard treatments of cesarean wound. Nonetheless, longer duration of the intervention might lead to a significant difference in the process of wound healing. Therefore, further studies with longer intervention periods are recommended to be conducted on the issue.
